# Opioid prescribing patterns at the end of life in an asian advanced cancer cohort: differences by palliative care consultation status

**DOI:** 10.1007/s00520-026-10621-1

**Published:** 2026-03-30

**Authors:** Hak Jun Kim, Jin-Ah Sim, Shin Hye Yoo

**Affiliations:** 1 Department of AI Convergence, Hallym University, Chuncheon, Republic of Korea; 2 Graduate School of Public Health and Healthcare Management, The Catholic University of Korea, Seoul, Republic of Korea; 3 Department of Population and Quantitative Health Science, UMass Chan Medical School, Worcester, MA USA; 4 Center for Palliative Care and Clinical Ethics, Seoul National University Hospital 101, Daehak-Ro, Jongno-Gu, Seoul, 03080 Republic of Korea; 5 Department of Human Systems Medicine, Seoul National University College of Medicine, Seoul, Republic of Korea

**Keywords:** Palliative care, Opioid analgesics, Neoplasms, Pain management

## Abstract

**Purpose:**

Opioid prescribing at the end of life varies widely across clinical contexts, yet real-world evidence from Asian advanced cancer populations remains limited. This study characterized end-of-life opioid prescribing patterns and examined differences according to palliative care consultation status.

**Methods:**

We conducted a retrospective cohort study using tertiary hospital clinical records linked to Korean National Health Insurance claims data. Patients with advanced cancer who received a palliative care consultation were matched 1:1 with those without consultation using propensity scores. Opioid prescribing during the last 30 days of life was evaluated, including time-specific patterns across three intervals (30–15, 14–8, and 7–0 days before death) and opioid strength (strong vs. weak).

**Results:**

Among 18,048 eligible patients, 3,742 were matched in each group. Opioid prescriptions were more common among patients who received palliative care, with the largest differences observed 30–15 days before death and narrowing toward the final week. Strong opioid use remained consistently higher in the palliative care group across all intervals, whereas weak opioid use showed minimal variation. Differences were most pronounced in older adults and in stomach, colorectal, and pancreatobiliary cancers.

**Conclusion:**

Patients who received a palliative care consultation showed different patterns of end-of-life opioid prescribing, especially for strong opioids. Understanding such variation may support ongoing efforts to improve symptom management near the end of life.

**Supplementary Information:**

The online version contains supplementary material available at 10.1007/s00520-026-10621-1.

## Introduction

Pain is among the most prevalent and distressing symptoms in patients with advanced cancer, requiring prompt and effective management. Inadequate control of cancer-related pain can lead to a significant decline in the quality of life [1, 2], unnecessary healthcare utilization, such as emergency department visits or hospitalizations [3, 4], and increased healthcare costs [5]. Opioid analgesics remain the mainstay of pharmacological treatments for moderate to severe cancer pain [6, 7]. However, growing concerns about opioid misuse and the broader opioid epidemic have contributed to more cautious prescribing practices in recent years [8–11], affecting both cancer and non-cancer populations [3, 12, 13]. Consequently, ensuring access to effective opioid therapy has become a central challenge in cancer pain management [13].

Despite clear clinical indications, opioids appear to be underused in certain patient groups, including older adults, patients with limited access to palliative care services, and those with specific cancer types associated with a more favorable prognosis [14–16]. Factors contributing to this underuse [9, 12–14, 17] include stigma, inadequate communication, limited patient and family education, and insufficient training. Palliative care (PC) teams often play a role in addressing these challenges by providing specialized symptom assessment, education, and support for complex decision making. Previous research from Europe [18, 19] and Japan [14] has reported higher use of strong opioids among patients who receive PC services.

However, significant knowledge gaps persist regarding how these prescribing trajectories evolve during the final weeks of life outside Western healthcare settings, including Asian contexts. Addressing these gaps is crucial, as identifying disparities in opioid access according to PC status can help clinicians identify unmet pain management needs and guide the timely integration of PC into routine oncology care. Ultimately, such evidence is vital for optimizing resource allocation within healthcare systems and, most importantly, ensuring that patients with advanced cancer achieve a more dignified end-of-life experience.

In this study, we characterized end-of-life opioid prescribing patterns among patients with advanced cancer and examined differences between those who did and did not receive a PC consultation.

## Methods

### Study design and population

This retrospective cohort study was conducted at a single tertiary academic hospital in South Korea using customized data derived by linking institutional electronic medical records with claims data from the Korean National Health Insurance Service (NHIS). The NHIS is a mandatory single-payer system that provides near-universal health coverage for the Korean population [20]. Opioid analgesics prescribed for cancer-related pain are reimbursed under the national insurance program, with minimal variation in coverage by age, income level, or region. This linkage structure allowed us to track opioid prescriptions across all healthcare settings including our institution and other hospitals throughout the patients’ care trajectories.

The study population included adult patients (aged ≥ 19 years) diagnosed with one of five major cancers: lung (International Classification of Diseases, 10th Revision [ICD-10] codes C33–C34), colorectal (C18–C20), gastric (C16), liver (C22), and pancreatobiliary cancers (C23–C25) and those who received care at a university-affiliated tertiary hospital located in the capital city between January 2018 and December 2022. All included patients were confirmed dead by June 22, 2023. The five cancer types were selected because they represent the most common causes of cancer-related death in Korea and account for a large proportion of patients with advanced disease requiring end-of-life care. Restricting the analysis to these major cancer types was intended to enhance clinical relevance and reduce heterogeneity related to disease trajectory and care patterns.

Patients were categorized into the PC or non-PC groups based on whether they received a formal PC consultation at the study institution. Specialty PC consultations were initiated through clinician referral and delivered by an interdisciplinary team consisting of physicians, nurses, and social workers; pharmacists were not routinely involved in the consultation process. Because the institution does not operate an inpatient hospice ward, the PC team provides consultation-based support only in outpatient or general ward settings; medication management after transfer to external hospice facilities is not included. The consultation process typically included comprehensive assessment of pain and other symptoms, review and adjustment of analgesic regimens including opioid titration when clinically indicated, patient and family education, and support for care planning and decision making. Patients referred from the emergency department or intensive care unit were excluded to ensure consistency in the clinical context and timing of referral. The timing and frequency of PC consultations were determined according to individual clinical needs and were not standardized. For the purpose of this study, exposure to PC was defined as receipt of at least one formal PC consultation.

### Outcomes and variables

All opioid prescription records in the database contain the following information: (1) opioid main component (e.g., fentanyl, morphine, oxycodone); (2) prescription date; (3) days of supply; (4) prescription type (inpatient vs. outpatient). All the records from both care settings were arranged by date of issuance to construct each patient's unique opioid exposure history throughout the end-of-life period. Opioid exposure was assessed within predefined time windows using binary indicators. For each time window, patients were considered exposed if they had received one or more opioid prescriptions during that period, regardless of the number of prescriptions, opioid types, or care settings involved. This operational definition enabled consistent measurement of opioid exposure across the study cohort.

The primary outcome was any opioid prescription during the last 30 days of life. This period was selected as the main observation because it is widely used in end-of-life research and represents a clinically meaningful timeframe for evaluating symptom management and care intensity. Secondary analyses examined opioid use during the last 14 and 7 days of life, as well as time-specific prescribing across three discrete intervals: 30–15 days before death (T1), 14–8 days (T2), and 7–0 days (T3). The three subintervals were defined to examine temporal changes in prescribing patterns as death approached. Use of strong opioids (morphine, fentanyl, oxycodone, tapentadol, nalbuphine, buprenorphine, hydromorphone, pethidine) and weak opioids (tramadol, codeine) was examined using independent binary indicators for each opioid class within each time window [6]. For example, a patient could be coded as having received strong opioids during the last 7 days of life, while not having received weak opioids during that same period.

### Statistical analysis

Propensity scores estimating the likelihood of receiving a PC consultation were generated using logistic regression including age, sex, cancer type, receipt of chemotherapy, non-cancer Charlson Comorbidity Index (CCI), income level, and region of residence. A 1:1 nearest-neighbor match without replacement was applied using a caliper of 0.25 standard deviations of the logit of the propensity score. Covariate balance was assessed using standardized mean differences, with values < 0.1 considered acceptable. Variables used in the matching were limited to baseline covariates to avoid incorporating time-varying or post-exposure factors. For example, care setting (inpatient versus outpatient) was not included as a matching variable because it represents a varying aspect according to time of care rather than a fixed characteristic.

Primary and secondary outcomes—including opioid use during the last 30, 14, and 7 days of life; time-specific use within the 30–15, 14–8, and 7–0 day intervals; and use of strong versus weak opioids—were analyzed in the matched cohort. Group differences were evaluated using chi-square or Fisher’s exact tests. Logistic regression models were fitted to obtain odds ratios (ORs) and 95% confidence intervals (CIs), adjusting for any residual imbalance.

Subgroup analyses were conducted in the full (unmatched) cohort to describe variation in prescribing patterns across clinical groups, as analyses within the matched cohort would have resulted in small subgroup sizes and unstable estimates. Separate multivariable logistic regression models were fitted for age, sex, and cancer type subgroups, adjusting for CCI, income, region, chemotherapy within three months of death, and metastatic lesions. Metastatic lesions were identified using ICD-10 codes for secondary malignant neoplasms (C79.5 as bone or bone marrow, C78.6 as peritoneum or retroperitoneum, C79.3 as brain or cerebral meninges, C78.0 as lung). The subgroup-defining variable was omitted from the respective model to avoid overadjustment.

All analyses were performed using SAS version 9.4. Statistical significance was defined as a two-sided p-value < 0.05.

### Ethics

This study was conducted in accordance with the Declaration of Helsinki. The Institutional Review Board of Seoul National University Hospital (approval no. H-2305–088–1431) approved the study. The requirement for informed consent was waived due to the study’s retrospective design. All analyses were conducted using de-identified data and adhered to the relevant data protection and privacy regulations.

## Results

### Selection of the study cohort and propensity score matching

Of the 18,089 patients with one of the five major cancer types who died between January 2018 and June 2023, 41 were excluded because of missing data (n = 23) or mismatched dates of death between institutional and NHIS records (n = 18). The final study population comprised 18,048 patients. Among these, 4,102 patients were initially assigned to the PC group and 13,946 to the non-PC group. After excluding those referred to PC from the emergency department or intensive care unit (n = 360), 3,742 patients remained in the PC group. Propensity score matching yielded 3,742 matched patients in the non-PC group (Supplementary Figure S1).

### Baseline characteristics before and after matching

In the unmatched cohort, baseline characteristics differed across several variables. In the unmatched cohort (Supplementary Table S1), patients in the PC group were younger on average (mean ± standard deviation, 66.5 ± 11.3 vs. 69.2 ± 11.7 years), more often female (35.8% vs. 31.5%), and more likely to reside in metropolitan areas (62.7% vs. 51.9%) compared to the non-PC group. Cancer type distribution also differed: the PC group had a higher proportion of lung cancer and lower proportions of liver and pancreatobiliary cancers. Metastatic lesions and chemotherapy use were more common in the PC group. After matching (Table 1), all covariates were well balanced with standardized mean differences < 0.1.

**Table 1:** Baseline characteristics of matched patients with advanced cancer

Variables	Total (n = 7484)	PC (n = 3742)	Matched non-PC (n = 3742)	P-value	SMD
Age (group)					
< 65	3090 (41.3)	1543 (41.2)	1547 (41.3)	0.972	0.006
65- < 75	2451 (32.8)	1223 (32.7)	1228 (32.8)		
≥ 75	1943 (26.0)	976 (26.1)	967 (25.8)		
Sex					
Male	4829 (64.5)	2401 (64.2)	2428 (64.9)	0.514	0.015
Female	2655 (35.5)	1341 (35.8)	1314 (35.1)		
Household income					
First quartile (lowest)	1667 (22.3)	832 (22.2)	835 (22.3)	0.918	0.016
Second quartile	1115 (14.9)	568 (15.2)	547 (14.6)		
Third quartile	1534 (20.5)	767 (20.5)	767 (20.5)		
Fourth quartile (highest)	3168 (42.3)	1575 (42.1)	1593 (42.6)		
Residence					
Metropolitan^*^	4699 (62.8)	2345 (62.7)	2354 (62.9)	0.830	0.005
Urban/suburban	2785 (37.2)	1397 (37.3)	1388 (37.1)		
Non-cancer CCI					
0	721 (9.6)	368 (9.8)	353 (9.4)	0.884	0.019
1	1774 (23.7)	883 (23.6)	891 (23.8)		
2	1898 (25.4)	957 (25.6)	941 (25.2)		
≥ 3	3091 (41.3)	1534 (41.0)	1557 (41.6)		
Cancer type					
Lung	2673 (35.7)	1345 (35.9)	1328 (35.5)	0.974	0.016
Stomach	996 (13.3)	491 (13.1)	505 (13.5)		
Colon	1051 (14.0)	531 (14.2)	520 (13.9)		
Liver	947 (12.7)	472 (12.6)	475 (12.7)		
Gallbladder/pancreas	1817 (24.3)	903 (24.1)	914 (24.4)		
Receipt of chemotherapy					
3 months prior to death	6894 (92.1)	3447 (92.1)	3447 (92.1)	1.000	<.0001

*PC* palliative care, *SMD* standardized mean difference, *CCI* Charlson comorbidity index

Data are presented as n (%), unless otherwise specified

^*^Metropolitan cities refers to Seoul, Incheon, Busan, Daegu, Ulsan, Daejeon, and Gwangju

### Association between PC consultation and time-specific opioid use

In the matched cohort, opioid prescriptions during the last 30 days of life were more frequent in the PC group than in the non-PC group (75.8% vs. 68.1%; OR, 1.47; 95% CI, 1.33–1.63; p < 0.0001). This difference was also observed at 14 days before death (48.6% vs. 44.5%; OR, 1.18; 95% CI, 1.08–1.30; p < 0.001), but not at 7 days (26.8% vs. 24.9%; OR, 1.10; 95% CI, 0.99–1.22; p = 0.065) (Table 2).

**Table 2:** Comparisons of any opioid use across overlapping time intervals at the end-of-life among matched patients

Time frame	PC (n = 3,742)	Matched non-PC (n = 3,742)	OR^*^ (95% CI)	P-value
1 month before death	2838 (75.8)	2549 (68.1)	1.47 (1.33, 1.63)	<.0001
2 weeks before death	1820 (48.6)	1664 (44.5)	1.18 (1.08, 1.30)	<.001
1 week before death	1002 (26.8)	932 (24.9)	1.10 (0.99, 1.22)	0.065

*PC* palliative care, *OR* odds ratio, *CI* confidence interval

Data are presented as n (%), unless otherwise specified

The end-of-life was defined as the final 30 days before death

^*^The non-PC group was used as the reference

**Fig. 1:**
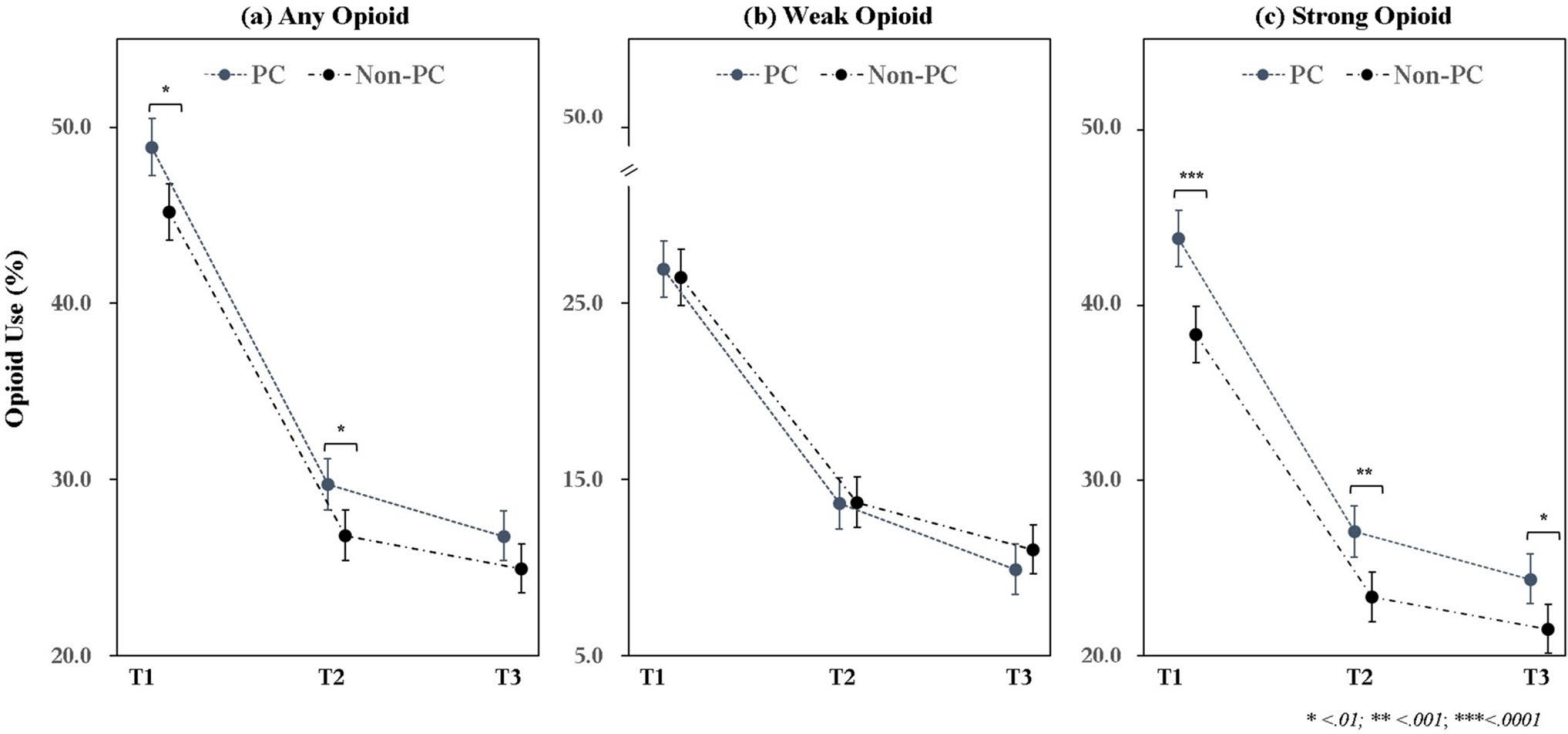
Comparisons of opioid use by strength at the end-of-life among matched patients. Each panel displays comparisons of the proportion of matched palliative care (PC) and non-PC patients who received opioids across three time intervals in the final 30 days before death. **a** Any opioid use; **b** Weak opioid use. **c** Strong opioid use. T1: 30 to 15 days before death. T2: 14 to 8 days before death. T3: 7 days before death. Error bars represent 95% confidence intervals. Significance levels are indicated as follows: * < 0.01, ** < 0.001, and *** < 0.0001

Across the three intervals (T1, T2, T3), the PC group had higher rates of opioid prescribing in T1 (48.9% vs. 45.2%; p = 0.001) and T2 (29.7% vs. 26.8%; p = 0.005), whereas the difference in T3 (26.8% vs. 24.9%; p = 0.065) was not statistically significant (Fig. 1). Strong opioid prescribing was higher in the PC group across all intervals—T1 (43.6% vs. 38.1%; p < 0.0001), T2 (26.9% vs. 23.1%; p < 0.001), and T3 (24.2% vs. 21.3%; p = 0.003)—while weak opioid use did not differ significantly at any interval.

### Opioid strength and agent-level differences between groups

Strong opioids were prescribed more frequently than weak opioids in both groups. During the last 30 days of life, 72.7% of patients in the PC group received at least one strong opioid, compared to 62.4% in the non-PC group (OR, 1.61; 95% CI, 1.46–1.77; p < 0.0001). Weak opioid use was also slightly more common in the PC group (54.9% vs. 52.4%; OR, 1.11; 95% CI, 1.01–1.21; p = 0.026) (Table 3). These findings are consistent with the time-specific trends shown in use of strong opioids (Fig. 1).

**Table 3:** Comparisons of opioid use by strength and type at the end-of-life among matched patients

Variables	PC (n = 3,742)	Matched non-PC (n = 3,742)	OR^*^ (95% CI)	P-value
Opioid Strength				
Strong	2722 (72.7)	2335 (62.4)	1.61 (1.46, 1.77)	<.0001
Weak	2056 (54.9)	1960 (52.4)	1.11 (1.01, 1.21)	0.026
Opioid Type				
Tramadol	1921 (51.3)	1818 (48.6)	1.12 (1.02, 1.22)	0.017
Codeine	366 (9.8)	496 (13.3)	0.71 (0.61, 0.82)	<.0001
Tapentadol	18 (0.5)	57 (1.5)	0.31 (0.18, 0.53)	<.0001
Morphine	2261 (60.4)	1743 (46.6)	1.75 (1.60, 1.92)	<.0001
Hydromorphone	26 (0.7)	31 (0.8)	0.84 (0.50, 1.41)	0.507
Oxycodone	1565 (41.8)	1318 (35.2)	1.32 (1.20, 1.45)	<.0001
Fentanyl	1822 (48.7)	1618 (43.2)	1.25 (1.14, 1.36)	<.0001
Nalbuphine	13 (0.3)	12 (0.3)	1.08 (0.49, 2.38)	0.841
Pethidine	979 (26.2)	1042 (27.8)	0.92 (0.83, 1.02)	0.101
Buprenorphine	35 (0.9)	69 (1.8)	0.50 (0.33, 0.76)	0.001

*PC* palliative care, *OR* odds ratio, *CI* confidence interval

Data are presented as n (%), unless otherwise specified

The end-of-life was defined as the final 30 days before death

^*^The non-PC group was used as the reference

Regarding specific opioid agents, morphine, tramadol, fentanyl, and oxycodone were most frequently prescribed in both groups during the last month of life. However, morphine (60.4% vs. 46.6%; OR, 1.75; 95% CI, 1.60–1.92; p < 0.0001), oxycodone (41.8% vs. 35.2%; OR, 1.32; 95% CI, 1.20–1.45; p < 0.0001), fentanyl (48.7% vs. 43.2%; OR, 1.25; 95% CI, 1.14–1.36; p < 0.0001), and tramadol (51.3% vs. 48.6%; OR, 1.12; 95% CI, 1.02–1.22; p = 0.017) were all more commonly prescribed in the PC group. In contrast, codeine, tapentadol, and buprenorphine were prescribed more frequently in the non-PC group (Table 3).

### Subgroup variation in the association between PC consultation and opioid use (full cohort analysis)

**Fig. 2:**
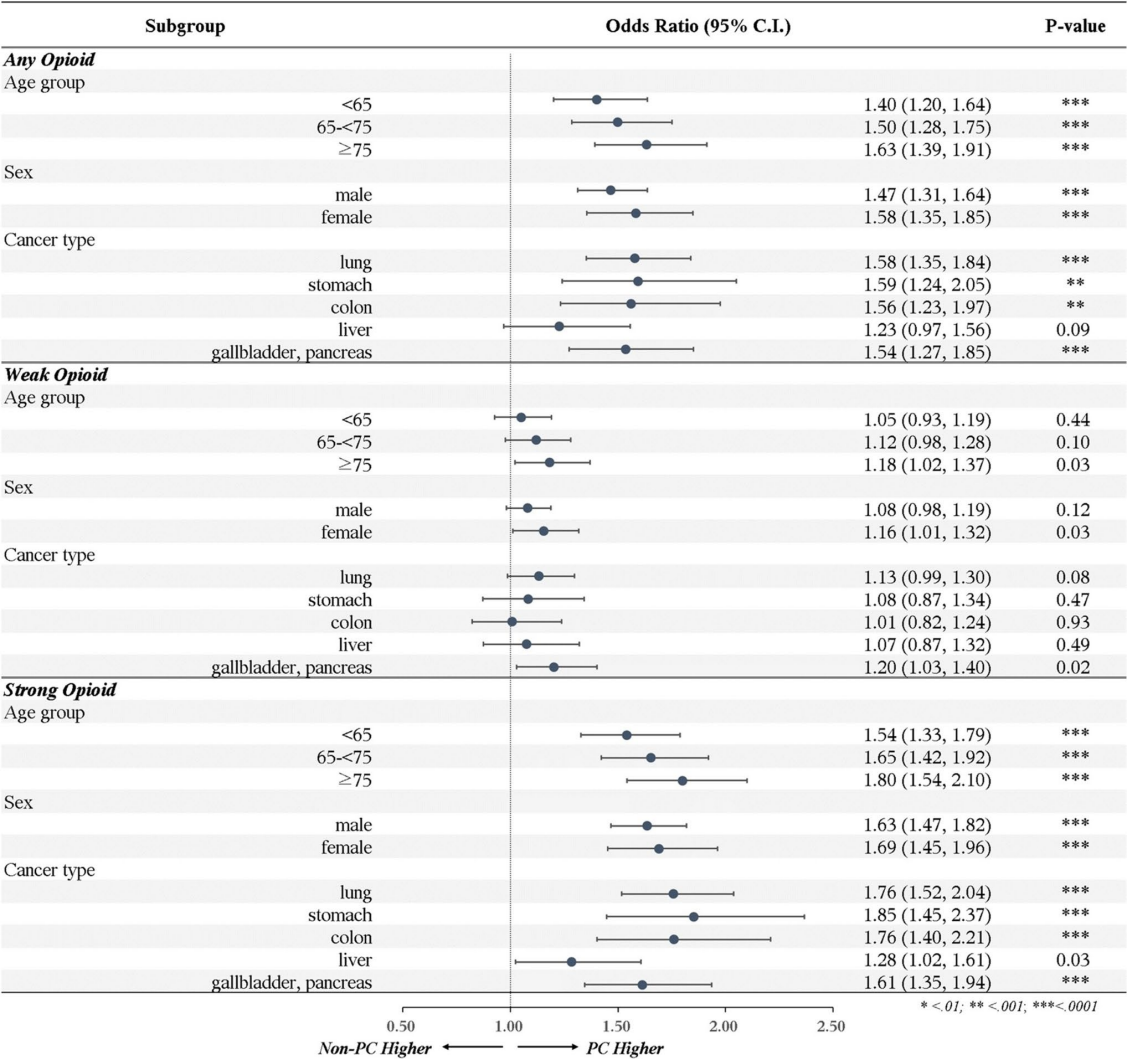
Subgroup comparisons of opioid use by type at the end-of-life among all patients. The unmatched full cohorts were analyzed in the final 30 days before death stratified by age group, sex, and cancer type. When subgrouping by each variable, the odds ratios were adjusted for the other two subgrouping variables as well as for common covariates, including household income, residence, non-cancer Charlson Comorbidity Index (CCI), receipt of chemotherapy (CTx), and metastatic lesions

Subgroup analyses in the full cohort evaluated variation by age, sex, and cancer type (Fig. 2). For any opioid use, an association with PC consultation was observed across all age groups, with higher aORs in older patients: aOR 1.40 (95% CI, 1.20–1.64) for patients aged < 65 years, 1.50 (1.28–1.75) for those aged 65–74 years, and 1.63 (1.39–1.91) for those aged ≥ 75 years (all p < 0.0001). Both male and female patients had higher opioid use associated with PC consultation (male: aOR 1.47, 95% CI 1.31–1.64; female: aOR 1.58, 95% CI 1.35–1.85). By cancer type, the association was significant for lung (aOR 1.58), stomach (aOR 1.59), colon (aOR 1.56), and pancreatobiliary cancers (aOR 1.54), but not for liver cancer (aOR 1.23, 95% CI 0.97–1.56; p = 0.089).

### Opioid type and timing relative to death: adjusted analyses in the full cohort

For weak opioid use, an association with PC consultation was observed only in patients aged ≥ 75 years (aOR 1.18, 95% CI 1.02–1.37; p = 0.025) and in female patients (aOR 1.16, 95% CI 1.01–1.32; p = 0.033), with no significant associations identified by cancer type (Fig. 2). For strong opioid use, adjusted odds ratios were higher across all subgroups, ranging from 1.54 to 1.80 across age groups, 1.63 for male and 1.69 for female patients, and 1.28 to 1.85 across cancer types. For liver cancer, the aOR was 1.28 (95% CI 1.02–1.61; p = 0.031), while associations for any and weak opioid use were not significant (Fig. 2).

In analyses stratified by time intervals before death (Supplementary Figures S2A–S2C), aORs for strong opioids were markedly higher in earlier intervals (T1) across all subgroups, while this pattern was solely observed in female patients for weak opioids. As patients who prescribed with strong opioids approached the final phase (T3), patterns became clearly distinct among certain subgroups, with significant estimates confirmed in older patients (≥ 75) and those with lung and colon cancers. In contrast, there were no significant subgroup differences for weak opioids at this time interval.

## Discussion

In this large cohort of patients with advanced cancer, PC consultations were associated with higher opioid prescribing, particularly for strong opioids, during the last month of life. This pattern was observed across time intervals, although prescribing during the final days was less distinct. These findings indicate that PC involvement may influence opioid use throughout the end-of-life period. Examination of temporal patterns, opioid strength, and subgroup differences suggests that differences between patients with and without PC consultation may reflect variations in the adequacy of pain management in routine clinical practice.

Although higher opioid use associated with PC consultation was observed across most intervals, the difference was smaller in the final week of life. This decline in prescribing near death was observed in both the PC and non-PC groups. Several factors may contribute to this pattern, including limited opportunities for medication adjustments late in the disease course [21], transitions to other care settings such as secondary hospitals, hospices, or long-term care facilities [22, 23]. Prior studies have also suggested that declining functional status in advanced disease stages may hinder oral medication administration [24, 25]. Similar decreases in prescribing activity during the final days of life have been described in studies of end-of-life care, suggesting that this pattern reflects the common clinical trajectory near death rather than differences related to PC involvement [12, 26].

A notable finding was the greater use of strong opioids among patients who received PC consultation. This observation aligns with previous evidence suggesting that PC may support timely escalation when stronger analgesia is clinically appropriate [19]. In contrast, weak opioid use showed minimal differences, consistent with broader concerns regarding their effectiveness [14, 27], ceiling effects, and adverse event profiles [28] in advanced cancer. These findings suggest that differences in prescribing patterns may relate more to clinical appropriateness than to overall increases in analgesic use [13, 19, 27, 29].

Subgroup analyses showed that the association between PC consultation and higher opioid use was present across age groups, with relatively larger effect estimates in older adults. These results are consistent with earlier reports documenting undertreatment of pain in older patients and may reflect enhanced support for decision-making, symptom assessment, and communication when PC is involved [12, 14, 22]. Slightly higher effect estimates in female patients may relate to variations in symptom expression or communication patterns, although clinical implications remain uncertain [24, 29–31].

Differences by cancer type were also observed. Associations were evident for lung, stomach, colorectal, and pancreatobiliary cancers, whereas estimates for liver cancer were smaller and not statistically significant for any and weak opioid prescribing, although associations for strong opioids persisted. Several factors may explain this discrepancy. Pain in liver cancer is often visceral and may be less persistent or severe than the neuropathic or bony pain observed in other cancers [32, 33]. Concerns regarding hepatic encephalopathy and impaired drug metabolism in advanced liver disease may prompt more cautious prescribing practices [33]. These considerations highlight the nuanced nature of pain management in liver cancer and the potential need for adapted approaches for symptom control in this group [27].

Morphine, oxycodone, and fentanyl were the most frequently prescribed strong opioids, and their use was more common among patients who underwent PC consultation. These patterns may reflect prescribing preferences in the study setting and the role of PC in facilitating the use of opioid formulations familiar to general clinicians. Although agent-level differences were not the primary focus of this study, they offer insight into medication choices in end-of-life care.

Overall, the findings suggest that PC consultation is associated with prescribing patterns consistent with established approaches to managing cancer-related pain [12, 19]. Given the high prevalence of moderate-to-severe pain near the end of life reported in previous studies [34, 35], lower opioid use among patients without PC consultation may reflect potential unmet needs in pain assessment rather than lower symptom burden [36]. Nevertheless, limited access to PC services and insufficient expertise among general clinicians may contribute to disparities in opioid prescriptions [12, 14, 24, 27, 29]. From a clinical perspective, timely PC involvement may help reduce potential undertreatment and support more appropriate pain control during the end-of-life period. Expanding access to PC and strengthening education in cancer pain management may help promote more consistent and appropriate prescribing [14, 24, 29].

This study has several limitations. First, as an observational study, causality cannot be inferred. While propensity score matching helped balance measured baseline characteristics between PC and non-PC groups, unmeasured confounding may remain. Most critically, we lacked data on baseline pain severity and symptom burden – key factors that likely influence both PC referral decisions and opioid prescribing patterns. Patients referred for PC may have had more severe or refractory symptoms, which could lead to increased opioid prescribing independent of PC involvement. This confounding would tend to overestimate the association between PC consultation and opioid use. Second, NHIS does not provide the entire drug code to prevent identification of pharmaceutical manufacturers; therefore, detailed dosing information was unavailable. Consequently, calculation of morphine milligram equivalents was not feasible since specific dosage amounts and routes of administration (e.g., oral, intravenous, transdermal) were restricted, which precluded assessment of dose escalation patterns, or prescribed doses relative to pain severity. Third, this study was conducted at a single tertiary hospital in Korea, and caution is warranted when generalizing the findings to other settings. Healthcare systems, socioeconomic conditions, access to opioid analgesics, and models of PC delivery vary substantially across countries, particularly within the diverse healthcare contexts of Asia, and these differences may influence prescribing patterns. Korea represents a high-income healthcare system with universal national insurance coverage and well-developed cancer care infrastructure; therefore, the findings may be most applicable to similar high-resource healthcare environments. Despite these limitations, the study has notable strengths, including a large cohort drawn from a comprehensive data linkage between institutional records and national insurance claims, which enabled population-level tracking of opioid prescriptions across all care settings. Additionally, our granular analysis across multiple end-of-life intervals provided detailed insights into how prescribing patterns evolved as death approached.

## Conclusions

In conclusion, PC consultation was associated with higher opioid prescribing, particularly strong opioids, during the last month of life. This pattern was observed across subgroups and time intervals, with smaller differences in the final days of life. These findings provide insight into opioid prescribing practices near the end of life and highlight patient groups in which differences were more pronounced, such as older adults and those with lung and gastrointestinal cancers.

## Supplementary Information

Below is the link to the electronic supplementary material.

Supplementary file1 (PDF 965 KB)

## Data Availability

The data supporting the findings of this study are not publicly available. Study participants were completely de-identified by National Health Insurance Service in Korea. The study protocol and statistical analysis plan can be shared upon reasonable request to the corresponding authors.
