# Supplementary material for: Opioid prescribing patterns at the end of life in an asian advanced cancer cohort: differences by palliative care consultation status
Source: Support Care Cancer. 2026 Mar 30;34(4):387. doi: 10.1007/s00520-026-10621-1 (PMC13035612; doi:10.1007/s00520-026-10621-1)
Supplement: Supplementary file 1 — Supplementary file1 (PDF 965 KB) [file 520_2026_10621_MOESM1_ESM.pdf]

Supplementary Table S1. Baseline Characteristics of Unmatched Cancer Patients according to Palliative Consultation

	Total (N = 17,688)	PC (N = 3,742)	Non-PC (N = 13,946)	P-value
**Age (years)**				
Mean ± sd	68.7 ± 11.7	66.5 ± 11.3	69.2 ± 11.7	<.0001
<65	6,154 (34.8)	1,543 (41.2)	4,611 (33.1)	
65-<75	5,498 (31.1)	1,223 (32.7)	4,275 (30.7)	
≥75	6,036 (34.1)	976 (26.1)	5,060 (36.3)	
**Sex**				
Male	11,960 (67.6)	2,401 (64.2)	9,559 (68.5)	<.0001
Female	5,728 (32.4)	1,341 (35.8)	4,387 (31.5)	
**Insurance type**				
National Health Insurance	16,869 (95.4)	3,582 (95.7)	13,287 (95.3)	0.25
Medicaid	819 (4.6)	160 (4.3)	659 (4.7)	
**Household income**				
First quartile (lowest)	3,958 (22.4)	832 (22.2)	3,126 (22.4)	0.72
Second quartile	2,600 (14.7)	568 (15.2)	2,032 (14.6)	
Third quartile	3,710 (21.0)	767 (20.5)	2,943 (21.1)	
Fourth quartile (highest)	7,420 (41.9)	1,575 (42.1)	5,845 (41.9)	
**Residence**				
Metropolitan*	9,586 (54.2)	2,345 (62.7)	7241 (51.9)	<.0001
Urban / Suburban	8,102 (45.8)	1,397 (37.3)	6,705 (48.1)	
**Non-cancer CCI**				
0	2,164 (12.2)	368 (9.8)	1,796 (12.9)	<.0001
1	4,045 (22.9)	883 (23.6)	3,162 (22.7)	
2	4,164 (23.5)	957 (25.6)	3,207 (23.0)	
≥3	7,315 (41.4)	1,534 (41.0)	5,781 (41.5)	
**Cancer type**				
Lung	4,362 (24.7)	1,345 (35.9)	3,017 (21.6)	<.0001
Stomach	2,204 (12.5)	491 (13.1)	1,713 (12.3)	
Colon	2,503 (14.2)	531 (14.2)	1,972 (14.1)	
Liver	4,004 (22.6)	472 (12.6)	3,532 (25.3)	
Gallbladder / Pancreas	4,615 (26.1)	903 (24.1)	3,712 (26.6)	
**Time from diagnosis to death (days) - Mean ± sd**	1125.2 ± 1252	1010.9 ± 1080	1155.9 ± 1293	<.0001
**Presence of metastatic lesion**				
Bone / Bone marrow	2,317 (13.1)	678 (18.1)	1,639 (11.8)	<.0001
Peritoneum	1,769 (10.0)	434 (11.6)	1,335 (9.6)	<.001
Brain / Cerebral meninges	1,647 (9.3)	585 (15.6)	1,062 (7.6)	<.0001
Lung	1,917 (10.8)	394 (10.5)	1,523 (10.9)	0.49
**Receipt of chemotherapy**				
3 months prior to death	15,890 (89.8)	3,447 (92.1)	12,443 (89.2)	<.0001

Abbreviation: palliative care, PC; standardized mean difference, SMD; Charson Comorbidity Index, CCI;
Data are presented as n (%), unless otherwise specified.
*Metropolitan cities refers to Seoul, Incheon, Busan, Daegu, Ulsan, Daejeon, and Gwangju.

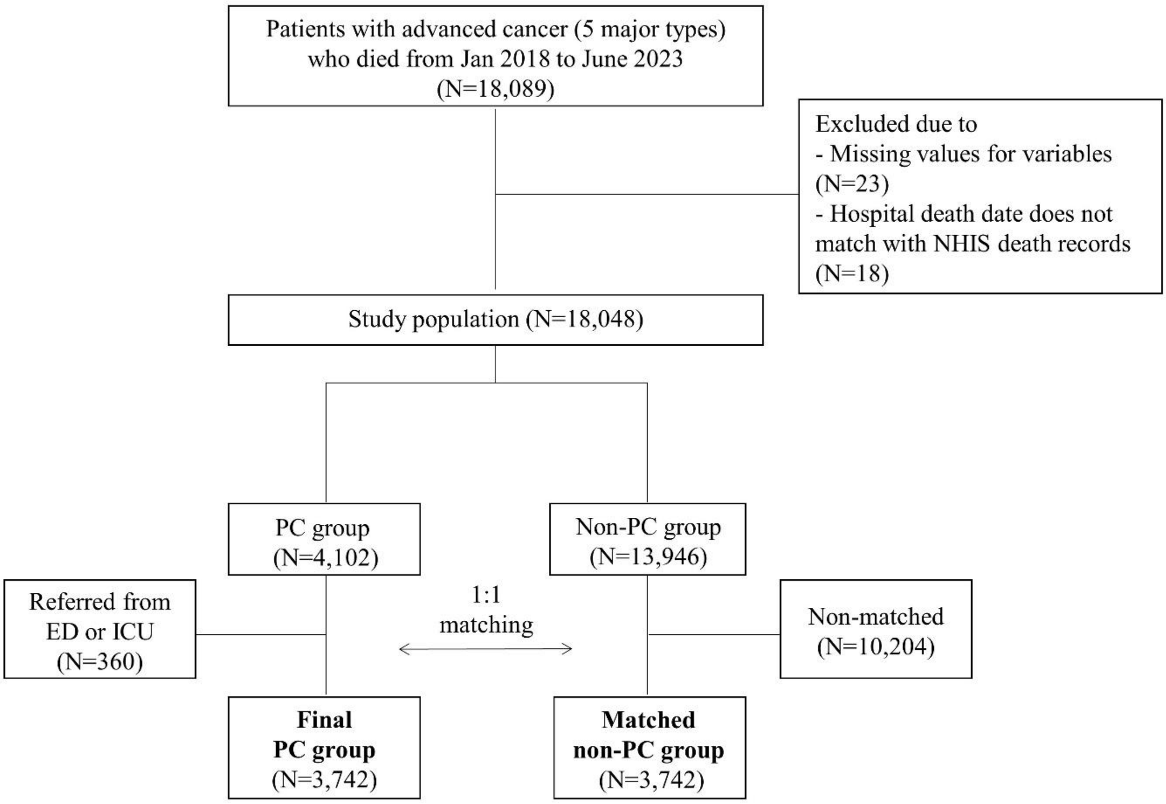

Supplementary Figure S1. Study flow chart

Subgroup	Odds Ratio (95% C.I.) - *any opioid use*	P-value
**T1:** *30 - 15 days before death*		
Age group		
<65	1.12 (1.00, 1.27)	0.06
65-<75	1.10 (0.96, 1.26)	0.16
≥75	1.23 (1.06, 1.43)	0.01
Sex		
male	1.08 (0.98, 1.19)	0.12
female	1.30 (1.14, 1.48)	***
Cancer type		
lung	1.08 (0.94, 1.24)	0.25
stomach	1.21 (0.98, 1.50)	0.08
colon	1.17 (0.95, 1.43)	0.14
liver	1.28 (1.05, 1.57)	0.02
gallbladder, pancreas	1.16 (0.99, 1.35)	0.06
**T2:** *14 - 8 days before death*		
Age group		
<65	1.06 (0.93, 1.21)	0.35
65-<75	1.21 (1.04, 1.40)	0.01
≥75	1.29 (1.09, 1.52)	*
Sex		
male	1.17 (1.06, 1.30)	*
female	1.15 (1.00, 1.32)	0.05
Cancer type		
lung	1.29 (1.11, 1.50)	**
stomach	1.24 (0.98, 1.56)	0.07
colon	1.13 (0.91, 1.42)	0.26
liver	0.95 (0.76, 1.19)	0.67
gallbladder, pancreas	1.14 (0.97, 1.33)	0.12
**T3:** *7 days before death to death*		
Age group		
<65	0.97 (0.85, 1.11)	0.62
65-<75	1.15 (0.99, 1.34)	0.06
≥75	1.34 (1.13, 1.58)	**
Sex		
male	1.17 (1.05, 1.30)	*
female	1.03 (0.89, 1.20)	0.66
Cancer type		
lung	1.34 (1.15, 1.56)	**
stomach	1.09 (0.86, 1.39)	0.46
colon	1.32 (1.06, 1.64)	0.01
liver	0.84 (0.67, 1.06)	0.15
gallbladder, pancreas	0.99 (0.83, 1.17)	0.88

** <.01; ** <.001; ***<.0001*

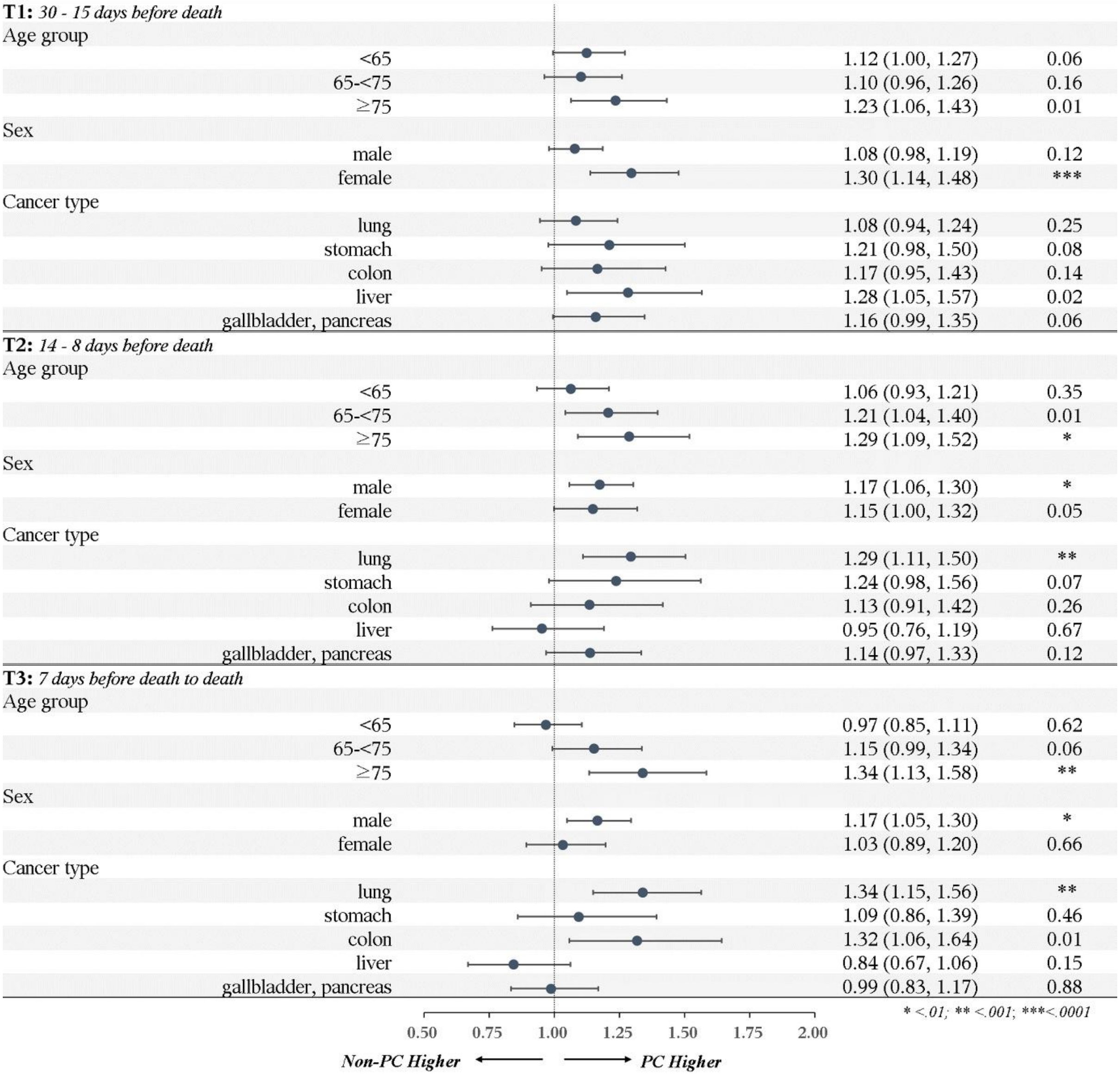

Supplementary Figure S2A. Subgroup Comparisons of Palliative Consultation in Any Opioid Use by Separate Time Intervals within 1 Month before Death

When subgrouping by each variable, odds ratios were adjusted for the other two subgrouping variables, as well as for common covariates including household income, residence, Charlson Comorbidity Index (CCI), receipt of chemotherapy (CTx), and metastatic lesions.

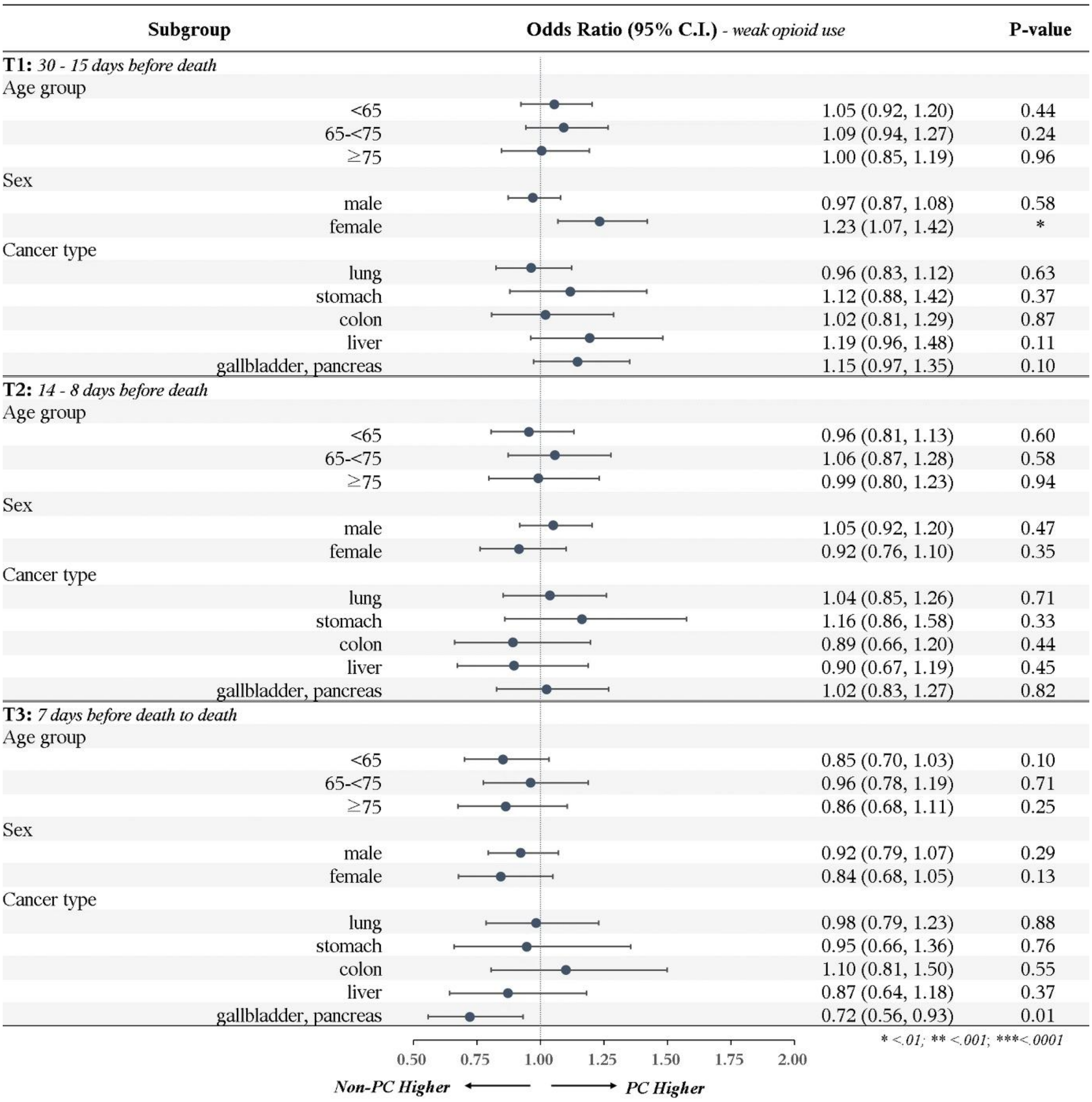

Supplementary Figure S2B. Subgroup Comparisons of Palliative Consultation in Weak Opioid Use by Separate Time Intervals within 1 Month before Death

When subgrouping by each variable, odds ratios were adjusted for the other two subgrouping variables, as well as for common covariates including household income, residence, Charlson Comorbidity Index (CCI), receipt of chemotherapy (CTx), and metastatic lesions.

Subgroup	Odds Ratio (95% C.I.) - *strong opioid use*	P-value
**T1:** *30 - 15 days before death*		
Age group		
<65	1.16 (1.03, 1.31)	0.01
65-<75	1.22 (1.06, 1.40)	*
≥75	1.41 (1.21, 1.64)	***
Sex		
male	1.17 (1.06, 1.29)	*
female	1.38 (1.21, 1.58)	***
Cancer type		
lung	1.22 (1.05, 1.40)	0.01
stomach	1.31 (1.05, 1.63)	0.02
colon	1.28 (1.04, 1.57)	0.02
liver	1.35 (1.10, 1.65)	*
gallbladder, pancreas	1.20 (1.03, 1.39)	0.02
**T2:** *14 - 8 days before death*		
Age group		
<65	1.18 (1.03, 1.34)	0.02
65-<75	1.24 (1.07, 1.45)	0.01
≥75	1.35 (1.13, 1.61)	*
Sex		
male	1.28 (1.14, 1.42)	***
female	1.18 (1.02, 1.36)	0.03
Cancer type		
lung	1.41 (1.20, 1.66)	***
stomach	1.36 (1.07, 1.74)	0.01
colon	1.24 (0.98, 1.56)	0.07
liver	1.00 (0.79, 1.27)	0.97
gallbladder, pancreas	1.16 (0.98, 1.36)	0.08
**T3:** *7 days before death to death*		
Age group		
<65	0.98 (0.86, 1.13)	0.81
65-<75	1.24 (1.06, 1.45)	0.01
≥75	1.52 (1.27, 1.82)	***
Sex		
male	1.25 (1.12, 1.40)	***
female	1.08 (0.92, 1.25)	0.35
Cancer type		
lung	1.47 (1.25, 1.73)	***
stomach	1.12 (0.87, 1.44)	0.37
colon	1.49 (1.18, 1.87)	**
liver	0.89 (0.70, 1.13)	0.33
gallbladder, pancreas	0.98 (0.83, 1.17)	0.86

** <.01; ** <.001; ***<.0001*

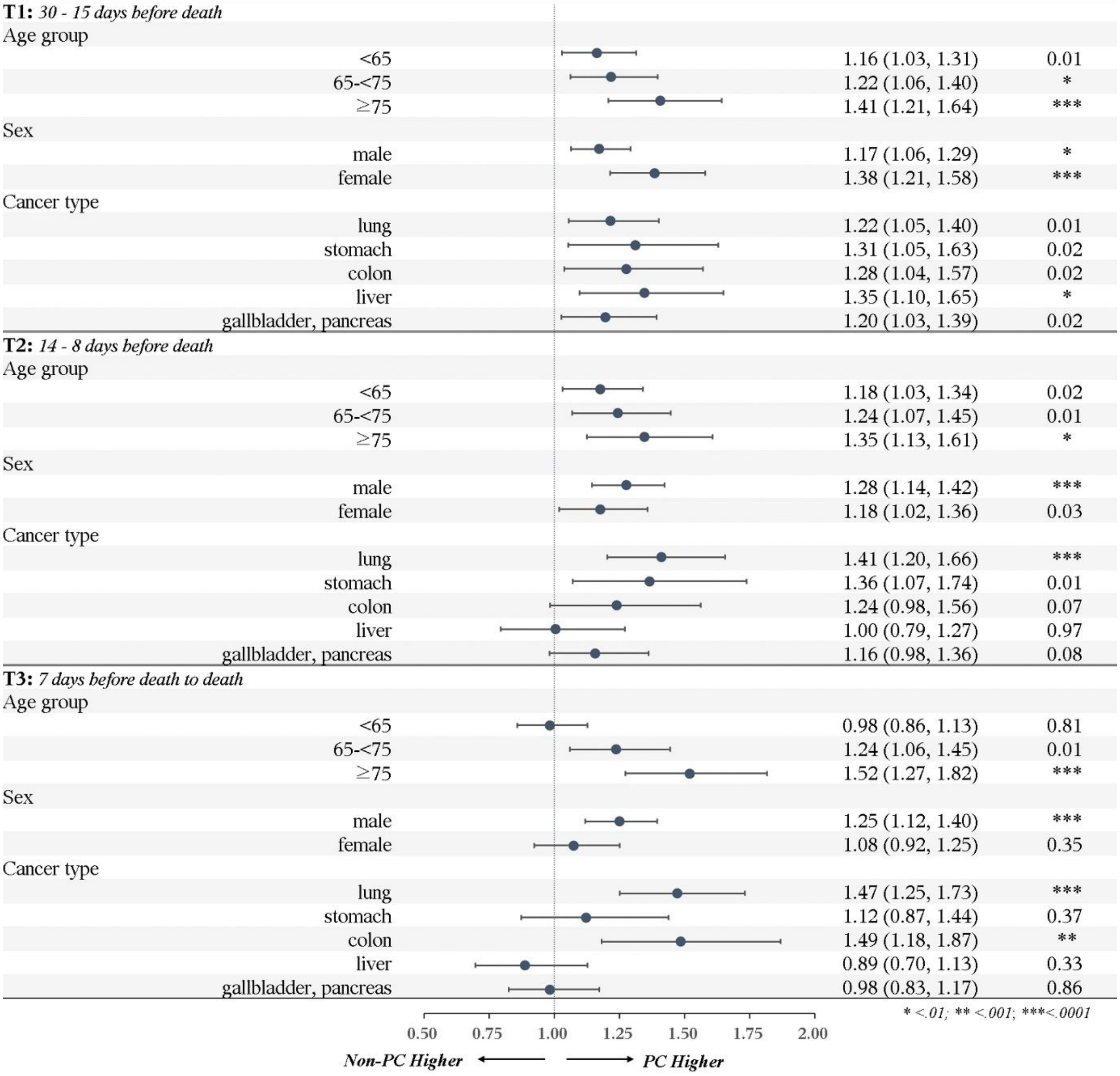

Supplementary Figure S2C. Subgroup Comparisons of Palliative Consultation in Strong Opioid Use by Separate Time Intervals within 1 Month before Death

When subgrouping by each variable, odds ratios were adjusted for the other two subgrouping variables, as well as for common covariates including household income, residence, Charlson Comorbidity Index (CCI), receipt of chemotherapy (CTx), and metastatic lesions.
